# Epidemiology of hip fracture and the development of FRAX in Ukraine

**DOI:** 10.1007/s11657-017-0343-2

**Published:** 2017-05-31

**Authors:** VV Povoroznyuk, NV Grygorieva, JA Kanis, McCloskey EV, H Johansson, NC Harvey, MO Korzh, SS Strafun, VM Vaida, FV Klymovytsky, RO Vlasenko, VS Forosenko

**Affiliations:** 1 State Institution, D. F. Chebotarev Institute of Gerontology NAMS Ukraine, Ukrainian Scientific Medical Center of Osteoporosis, Kyiv, Ukraine; 2 Centre for Metabolic Bone Diseases, University of Sheffield, S10 2RX, Sheffield, UK; 3 Institute for Health and Aging, Catholic University of Australia, Melbourne, Australia; 4 Centre for Integrated Research in Musculoskeletal Ageing (CIMA), Mellanby Centre for Bone Research, University of Sheffield, Sheffield, UK; 5 MRC Lifecourse Epidemiology Unit, University of Southampton, Southampton, SO16 6YD UK; 6 NIHR Southampton Biomedical Research Centre, University of Southampton and University Hospital Southampton NHS Foundation Trust, Tremona Road, Southampton, UK

**Keywords:** Epidemiology, Hip fractures, Ukraine, FRAX, Austria

## Abstract

***Summary*:**

A country-specific FRAX model has been developed for the Ukraine to replace the Austrian model hitherto used. Comparison of the Austrian and Ukrainian models indicated that the former markedly overestimated fracture probability whilst correctly stratifying risk.

**Introduction:**

FRAX has been used to estimate osteoporotic fracture risk since 2009. Rather than using a surrogate model, the Austrian version of FRAX was adopted for clinical practice. Since then, data have become available on hip fracture incidence in the Ukraine.

**Methods:**

The incidence of hip fracture was computed from three regional estimates and used to construct a country-specific FRAX model for the Ukraine. The model characteristics were compared with those of the Austrian FRAX model, previously used in Ukraine by using all combinations of six risk factors and eight values of BMD (total number of combinations =512).

**Results:**

The relationship between the probabilities of a major fracture derived from the two versions of FRAX indicated a close correlation between the two estimates (*r* > 0.95). The Ukrainian version, however, gave markedly lower probabilities than the Austrian model at all ages. For a major osteoporotic fracture, the median probability was lower by 25% at age 50 years and the difference increased with age. At the age of 60, 70 and 80 years, the median value was lower by 30, 53 and 65%, respectively. Similar findings were observed for men and for hip fracture.

**Conclusion:**

The Ukrainian FRAX model should enhance accuracy of determining fracture probability among the Ukrainian population and help to guide decisions about treatment. The study also indicates that the use of surrogate FRAX models or models from other countries, whilst correctly stratifying risk, may markedly over or underestimate the absolute fracture probability.

## Introduction

FRAX® is a computer-based algorithm developed by the former World Health Organization Collaborating Centre for Metabolic Bone Diseases and first released in 2008. This algorithm calculates fracture probability from clinical risk factors in women and men [1, 2]. The output of FRAX is the 10-year probability of a major osteoporotic fracture (hip, clinical spine, humerus or wrist fracture) and the 10-year probability of hip fracture. Probability is calculated from age, body mass index (BMI) and dichotomized risk factors comprising prior fragility fracture, parental history of hip fracture, current tobacco smoking, long-term oral glucocorticoid use, rheumatoid arthritis, other causes of secondary osteoporosis and excessive alcohol consumption. Femoral neck BMD can be optionally input to enhance fracture risk prediction [3].

The risk of hip fracture and probably of other osteoporotic fractures varies significantly around the world [4]. The difference in incidence between countries is much greater than the difference in incidence between sexes within a country. Indeed, greater than tenfold differences in hip fracture incidence have been reported in different countries. For this reason, FRAX models are calibrated for each country dependent on the epidemiology of death and fracture (most usually hip fracture). To date, FRAX models are available for 63 countries (http://www.shef.ac.uk/FRAX) covering more than 80% of the world population [5].

All the required information to build a FRAX model is not available in all countries. In such cases, the use of a surrogate model has been proposed [6] using the death rate of the index country and the fracture rate of a country thought to be similar to the index country in terms of fracture risk. Examples include Sri Lanka, India [7, 8] and until recently, Armenia. The Ukrainian Scientific Medical Centre on Osteoporosis Problems (Kiev) has used FRAX to estimate the osteoporotic fracture risk since 2009 [9]. Rather than using a surrogate model, the Austrian version of FRAX was adopted for clinical practice [10]. Since then, data have become available on hip fracture incidence in the Ukraine. The aims of the present study were to develop the Ukrainian FRAX model according to the age- and sex-specific hip fracture rates in Ukraine and to compare this with the Austrian FRAX model currently recommended for Ukraine.

## Methods

The development and validation of FRAX have been extensively described [1, 2]. The risk factors used were based on a systematic series of meta-analyses of population based cohorts worldwide and validated in independent cohorts with over 1 million patient-years of follow-up. The construct of the FRAX model for Ukraine required the beta coefficients of risk factors in the original FRAX model, and the incidence of hip fracture and death for Ukraine. The death hazard for 2009 was taken from the data base of the United Nations [11].

### Cohorts

The risk of hip fracture was calculated from two studies at three regional sites in Ukraine (Vinnitsa city (1997–2002), STOP-Study (Uzhgorod city and Vinnitsa area, 2011–2012). The results of these studies have been reported elsewhere [12, 13]. In brief, in the Vinnitsa city study, cases of hip fracture were identified retrospectively over a 6-year period (from 01.01.1997 to 31.12.2002) in men and women aged 50 years or more. The second study (Study of the prevalence of Osteoporotic fractures in Ukrainian Population—STOP-Study), organized by the Ukrainian Association of Osteoporosis, gathered retrospective information over 2 years (01.01.2011 to 31.12.2012) in two regions—Uzhhorod city and Vinnitsa area excluding Vinnitsa city.

### Ascertainment of incident fractures

All data were retrieved from multiple sources (the records of the ambulance service, city and district hospitals and outpatient departments). All instances of double-counting corresponding to multiple admissions for the same fracture were deleted before analysis. Cases were defined as patients (aged 40 years or more) who were identified with hip fracture (ICD10 code S72.0 [femoral neck], S72.1 [trochanter], S72.2 [subtrochanter]) irrespective of the level of trauma, but cases associated with neoplasia were excluded. We excluded patients with a code S72.9 (unspecified site of femoral fracture) except where a surgical procedure indicated surgery on the hip. Patients who had sustained a hip fracture in the previous year at the same site were excluded as were patients with multiple admissions for the same fracture in the index year. We included patients with a hip fracture, who used the ambulance service but refused hospitalization and treatment later. We included hip fracture cases irrespective of region or country of origin.

Cases were ascertained from the age of 40 years since this is the lower age limit used in FRAX. Incidence rates were estimated as the number of men and women in 5-year age intervals with one hip fracture in the year divided by the age- and sex-specific population of each catchment using government estimates for the same year. Data from the three regional studies were amalgamated weighted by catchment population. For the purposes of FRAX, data were smoothed using piecewise linear regression on log-transformed incidence rates by age with a breakpoint at 67 years of age. Thereafter, the exponent was included in the FRAX model so that risk could be calculated at any specific age rather than in 5-year intervals.

### Comparison of Ukrainian and Austrian FRAX models

For the purpose of comparing the Ukrainian and the Austrian models, probabilities of a major osteoporotic fracture (hip, clinical spine, forearm and humeral fractures) and of hip fracture alone, were computed in men and women at ages 50, 60, 70 and 80 years for all possible combinations of clinical risk factors at BMD T-scores between 0 and −3.5 SD in 0.5 SD steps with a BMI set to 25 kg/m^2^ [14]. Thus, we considered all combinations of six risk factors and eight values of BMD giving a total number of combinations of 512. Note that this was not a population simulation, but an array of all possible combinations. The correlation between the Austrian and Ukrainian fracture probabilities was examined by piecewise linear regression with knots at probabilities of 50 and 70% for the Austrian probabilities of a major osteoporotic fracture and at 5 and 20% for hip fracture using the same model. The reason for using knots at different probabilities for the two outcomes was because of the difference in the distribution of probabilities. Tabular data compared probabilities with the two versions at the 10th, 50th (median) and 90th percentile of the distribution of the Austrian model. Differences in the Ukrainian version at these percentiles were expressed as 95% tolerance intervals (TI).

In order to compare Ukrainian hip fracture probabilities with adjacent countries with a FRAX model and Austria, the remaining lifetime probability of hip fracture and major osteoporotic fracture from the age of 50 years was calculated for men and women, as described by Kanis et al. [15, 16].

## Results

**Fig. 1:**
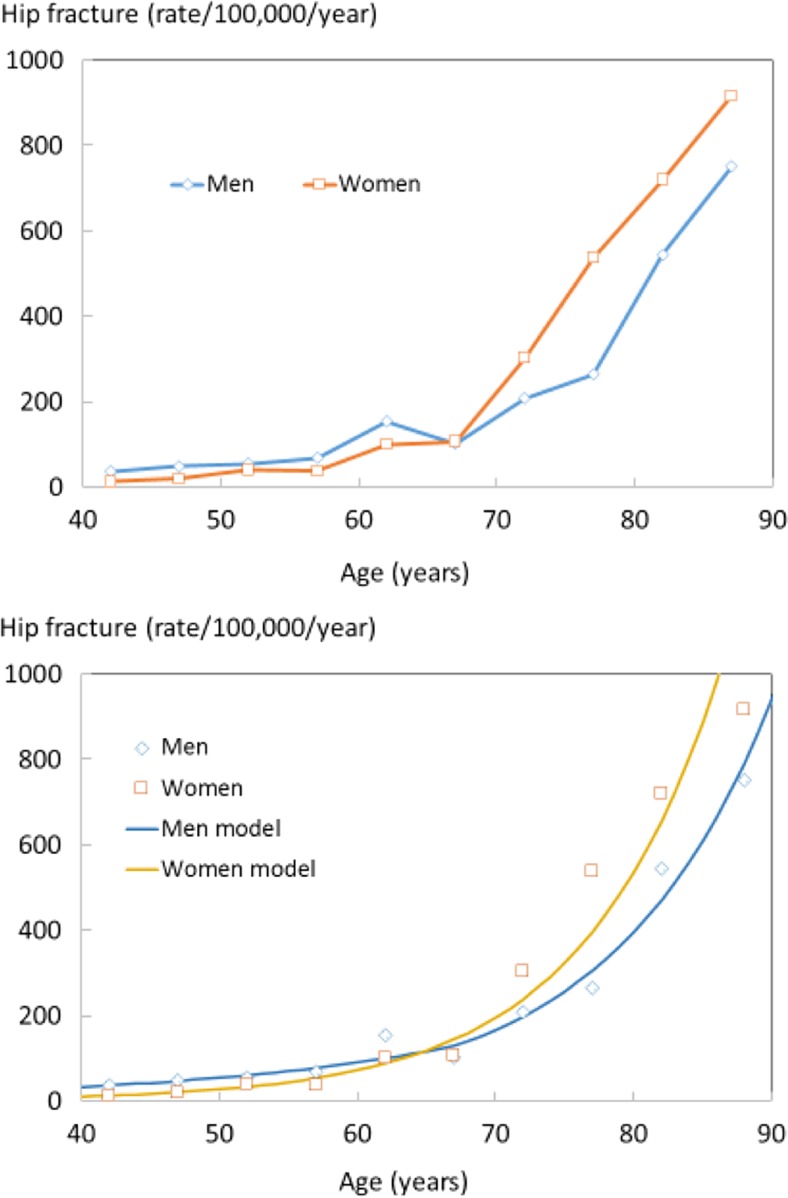
Observed Ukrainian age- and gender-stratified incidence of hip fracture (*upper panel*) and comparison of observed data (*symbols*) and 1-year incidence rates derived from piecewise linear regression (*lower panel*)

Figure 1 shows 1-year age- and gender-stratified incidence rates of hip fracture for the Ukraine from the three regional studies, as well as the incidence of hip fractures, based on the linear regression. Hip fracture incidence was lowest in patients aged 40–54 years and increased progressively with age. Below the age of 65 years, hip fracture incidence was higher in men than in women but, above this age the incidence increased more markedly in women compared to men.

### Fracture probability

**Fig. 2:**
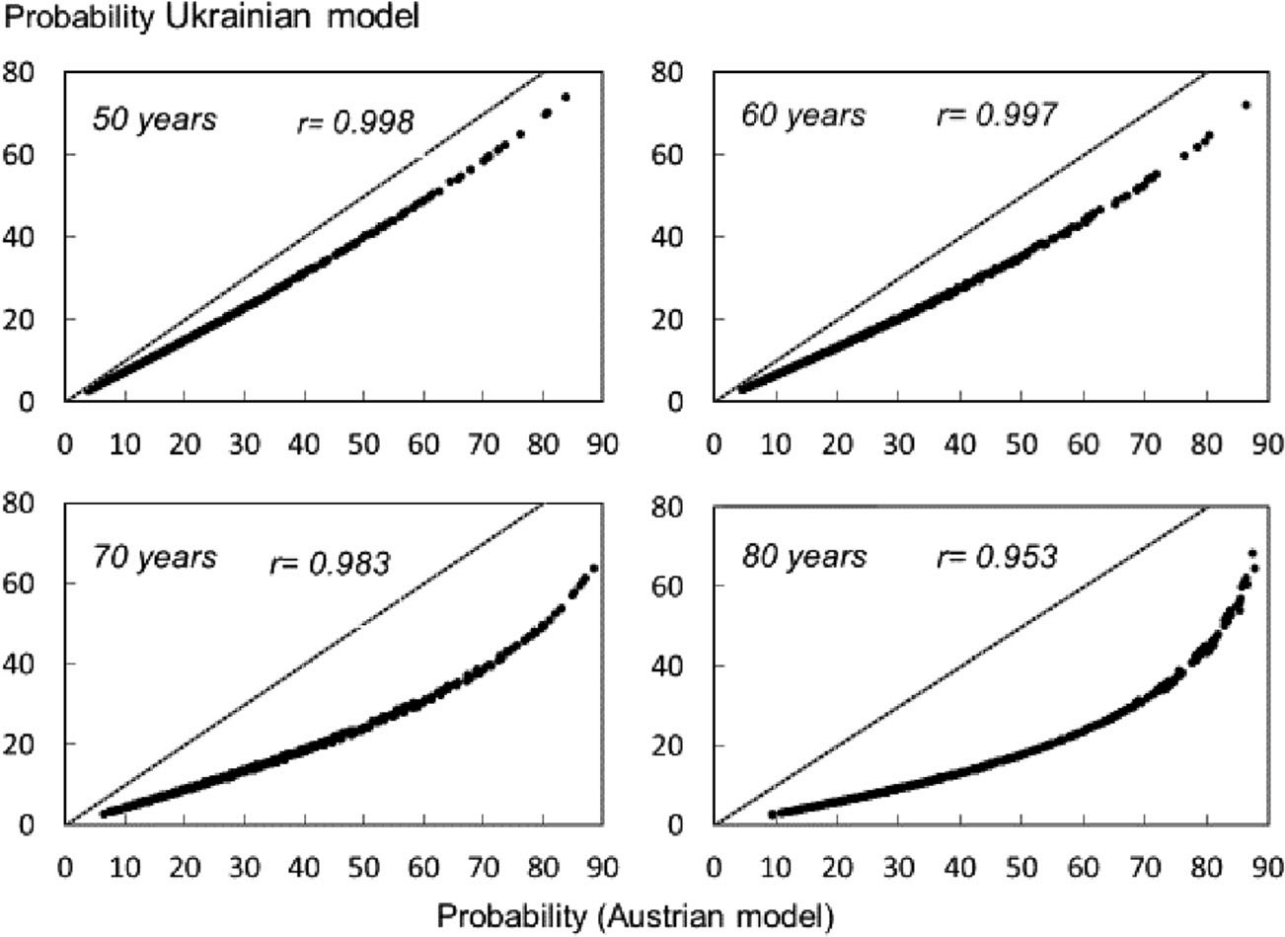
Comparison of 10-year probability of a major osteoporotic fracture using the Austrian FRAX tool applied to the Ukrainian female population and the Ukrainian tool for multiple clinical scenarios. The *diagonal dashed line* shows the line of identity

The relationship between the probabilities of a major fracture derived from the two versions of FRAX is shown for women aged 50 to 80 years in Fig. 2. At all ages, there was a close correlation between the two estimates (*r* > 0.95). The Ukrainian version gave lower probabilities than the Austrian model at all ages. The median value was lower by 25% at the age 50 years and the difference increased with age. At the age of 60, 70 and 80 years, the median value was lower by 30, 53 and 65%, respectively.

In the case of hip fracture, there was also a close correlation between the two estimates (*r* > 0.95) at all ages. The Ukrainian version gave lower estimates than the Austrian model at all ages and by a proportion that increased with age (Table 1). In men, the effect of the revision was qualitatively similar to that in women (Table 2).

**Table 1:** Probability (%) of a major osteoporotic fracture (MOF) or a hip fracture (with 95% tolerance intervals; TI) in women at the percentiles of the probability distribution (Austrian version) by age

	Percentile						*r* value
	10		50		90		
Age	Austria	Ukraine (95% TI)	Austria	Ukraine (95% TI)	Austria	Ukraine (95% TI)	
MOF							
50	8	6 (5–6)	20	15 (15–16)	49	38 (38–39)	0.998
60	10	6 (6–7)	23	16 (15–16)	51	36 (35–36)	0.997
70	13	5 (4–6)	30	14 (13–15)	65	35 (33–36)	0.983
80	19	5 (4–7)	43	15 (13–16)	76	40 (38–42)	0.953
Hip							
50	0.4	0.2 (0.0–0.5)	4	3 (3–3)	31	24 (24–25)	0.999
60	0.7	0.3 (0.0–0.7)	5	3 (3–4)	29	19 (19–20)	0.999
70	2.3	0.6 (0.0–1.5)	12	5 (4–6)	51	24 (23–4)	0.987
80	6.3	1.4 (0.0–2.9)	28	9 (7–11)	71	32 (30–34)	0.956

**Table 2:** Probability (%) of a major osteoporotic fracture (MOF) or a hip fracture (with 95% tolerance intervals; TI) in men at the percentiles of the probability distribution (Austrian version) by age

Age	Percentile						*r* value
	10		50		90		
	Austria	Ukraine (95% TI)	Austria	Ukraine (95% TI)	Austria	Ukraine (95% TI)	
MOF							
50	7	4 (3–6)	19	12 (11–14)	53	36 (34–37)	0.997
60	8	4 (3–5)	19	11 (10–12)	46	26 (25–27)	0.996
70	9	3 (2–4)	22	9 (8–9)	49	22 (21–22)	0.988
80	12	3 (2–4)	28	9 (8–10)	57	24 (23–25)	0.975
Hip							
50	0.7	0.3 (0.0–1.2)	6	4 (3–5)	40	26 (25–27)	0.998
60	1.1	0.5 (0.0–1.1)	6	3 (2–4)	31	17 (16–17)	0.997
70	2.6	0.6 (0.0–1.5)	12	5 (4–6)	42	18 (17–19)	0.989
80	5.7	0.9 (0.0–2.1)	21	7 (5–8)	54	21 (20–22)	0.975

The large disparities between Austrian and Ukrainian models were reflected in differences in the lifetime probabilities of a hip fracture from the age of 50 years. In Austria, this was 19.6% in women and 8.2% in men. The equivalent probabilities in Ukraine were 5.4 and 2.8%, respectively.

## Discussion

In this study, we documented the incidence of hip fracture in the Ukraine. The incidence of hip fracture was used to populate a country-specific FRAX tool to compute the 10-year probabilities of hip and major osteoporotic fracture. The new model can now replace the Austrian model used since 2009. In brief, the Ukrainian model provided substantially lower estimates of fracture probability at all ages. Importantly, the country-specific model had little impact on the categorization of risk, since the revisions did not change the rank order of fracture probability. In the clinical scenarios presented in this paper, the correlation coefficients between the Austrian and authentic versions for fracture probability exceeded 0.95 at all ages and in men as well as women. Thus, an individual at the 90th percentile of risk with the Austrian tool would still be very close to the 90th percentile of risk using the Ukrainian FRAX tool. For this reason, the consequences of using country-specific tools reside in the absolute number generated and not in the rank order of risk. The same phenomenon has been observed in FRAX revisions [14]. This is of little consequence to the management of patients or the interpretation of clinical studies. There is a useful analogy with the different DXA devices available, where a substantial difference in femoral neck BMD is seen between Hologic and Lunar machines, but the T-score derived from these is more or less identical [17]. However, difficulties arise when fracture probabilities are used in health economic analysis to inform practice guidelines or devise intervention thresholds.

It is evident that the use of the Austrian model grossly overestimated FRAX probabilities in Ukraine. Where FRAX models are not available for a specific country, the International Society for Clinical Densitometry and International Osteoporosis Foundation recommend the use of surrogate FRAX models using the fracture risk of a neighbouring country together with the death risk of the index country [6]. Ukraine, situated in Eastern Europe, shares borders with the Russian Federation to the east and north-east, and with Belarus towards the north-west border. Hungary, Slovakia and Poland are to its west; Romania and Moldova share its south-west border. A comparison of probabilities of the Ukrainian model with the Austrian model is given in Table 3 together with neighbouring counties. The probabilities were lower than in neighbouring countries with Poland being the closest. In view of the disparate results, the adoption of a surrogate country (rather than the entire Austrian model) might have given equally misleading results.

**Table 3:** Ten-year probability of major osteoporotic fracture (MOF) and hip fracture in men and women aged 65 years with a prior fragility fracture. (Body mass index set to 25 g/m^2^)

Country	Men	Hip fracture	Women	Hip fracture
	MOF		MOF	
Austria	9.7	2.5	17	4.3
Belarus	3.1	0.9	6.2	1.8
Hungary	6.2	1.6	12	3.1
Moldova	9.3	2.5	17	4.7
Poland	4.5	1.2	8.3	2.2
Romania	5.2	1.5	9.5	2.6
Russia	9.2	1.3	18	2.6
Slovakia	9.1	2.4	17	4.3
Ukraine	4.6	1.2	8.8	2.3

Ideally, FRAX models should use fracture rates for the whole country [4], whereas the present study sampled fracture rates from three regions (two of 24 provinces) representing only about 1% of the total population aged 40 years or more. It is well established that there are regional variations in hip fracture rates within countries [18–21]. Indeed, regional differences in hip fracture incidence have been reported using common methodology with the higher rates in urban communities in several countries [20–24] but, given the absence of national registers, we had to rely on the regional estimates. The situation is not unique and regional estimates have also been used to create FRAX models for Brazil [25–30].

A limitation of the present study is that we were not able to collect data on fracture at sites other than the hip. For this reason, the FRAX model relied on hip fracture rates to estimate the incidence of a major osteoporotic fracture. For this purpose, it is assumed that the ratio of hip fracture incidence to other FRAX outcomes (clinical spine, distal forearm and proximal humerus) is the same in the index country as that documented in Sweden. The ratios for Sweden were derived using national hip fracture data for Sweden and data from Malmo for the other fracture outcomes [31]. Despite many studies that have examined the incidence of fractures by age and sex, there are problems in defining the pattern of fractures in different countries. The available evidence indicates that the incidence of major fractures can be reasonably predicted from the incidence of hip fracture [31–33].

With these caveats, a country-specific FRAX model has been developed for the Ukraine which can now replace the Austrian model hitherto used. This model should enhance accuracy of determining fracture probability among the Ukrainian population and help to guide decisions about treatment. The study also indicates that the use of surrogate FRAX models or models from other countries, whilst correctly stratifying risk, may markedly over or underestimate the absolute fracture probability.
